# Microfluidic-based virus detection methods for respiratory diseases

**DOI:** 10.1007/s42247-021-00169-7

**Published:** 2021-03-25

**Authors:** E. Alperay Tarim, Betul Karakuzu, Cemre Oksuz, Oyku Sarigil, Melike Kizilkaya, Mahmoud Khatib A. A. Al-Ruweidi, Huseyin Cagatay Yalcin, Engin Ozcivici, H. Cumhur Tekin

**Affiliations:** 1 Department of Bioengineering, Izmir Institute of Technology, Urla, Izmir, Turkey; 2 Biomedical Research Center, Qatar University, Doha, Qatar; 3 METU MEMS Center, Ankara, Turkey

**Keywords:** Microfluidic, Respiratory disease, Viral pathogen, Virus Detection, Biosensors,, COVID-19

## Abstract

With the recent SARS-CoV-2 outbreak, the importance of rapid and direct detection of respiratory disease viruses has been well recognized. The detection of these viruses with novel technologies is vital in timely prevention and treatment strategies for epidemics and pandemics. Respiratory viruses can be detected from saliva, swab samples, nasal fluid, and blood, and collected samples can be analyzed by various techniques. Conventional methods for virus detection are based on techniques relying on cell culture, antigen-antibody interactions, and nucleic acids. However, these methods require trained personnel as well as expensive equipment. Microfluidic technologies, on the other hand, are one of the most accurate and specific methods to directly detect respiratory tract viruses. During viral infections, the production of detectable amounts of relevant antibodies takes a few days to weeks, hampering the aim of prevention. Alternatively, nucleic acid–based methods can directly detect the virus-specific RNA or DNA region, even before the immune response. There are numerous methods to detect respiratory viruses, but direct detection techniques have higher specificity and sensitivity than other techniques. This review aims to summarize the methods and technologies developed for microfluidic-based direct detection of viruses that cause respiratory infection using different detection techniques. Microfluidics enables the use of minimal sample volumes and thereby leading to a time, cost, and labor effective operation. Microfluidic-based detection technologies provide affordable, portable, rapid, and sensitive analysis of intact virus or virus genetic material, which is very important in pandemic and epidemic events to control outbreaks with an effective diagnosis.

## Introduction

Respiratory tract infections have been a great danger for children, adults, and elders for years. Influenza A and B, parainfluenza, adenovirus, respiratory syncytial virus, human metapneumovirus, human rhinoviruses, Enterovirus 71, bocavirus, and coronavirus are examples that can cause respiratory tract infections [1]. According to the World Health Organization (WHO) estimations, 1.9 million children die each year due to acute respiratory infections [2]. Based on its prevalence, respiratory tract infections are the sixth leading cause of mortality [3]. The coronavirus variants previously appeared as SARS-CoV-1 and MERS-CoV, and later emerged in 2019 in China as SARS-CoV-2 and spread worldwide within months [4]. Recent outbreaks of SARS-CoV-2 cause more than 1.5 million deaths as of 2020 December [5]. This virus can be transmitted both directly such as saliva and secretion droplets and indirectly from object surface [6]. Infected people show symptoms such as fever, cough, shortness of breath, fatigue, loss of taste or smell, headache, runny nose, and diarrhea [7]. According to evidence related to SARS-CoV-2, symptoms appear after approximately 5.2 days and the virus can cause hemorrhagic and immunologic responses that can affect multiple organs [8]. Long-term consequences of SARS-CoV-2 including neuropathy and decreased lung capacity are still unknown, but it will be enlightened by ongoing studies [9, 10]. It is difficult to distinguish SARS-CoV-2 and flu from each other because some symptoms are similar. Due to the high transmission rate of SARS-CoV-2 (R0: 1.4–5.5) and similar symptoms of SARS-CoV-2 with other respiratory viruses, early and specific diagnosis is required [11].

Microfluidic systems can be used in a wide range of areas in biotechnology such as detection, separation, and mixing, and therefore offer cutting-edge applications for the diagnosis and detection of diseases [12, 13]. Microfluidics including components such as pumps, actuators, and valves are miniature technologies that offer easy-to-use, efficient, and specific detection for biological agents [14, 15]. Moreover, microfluidic technologies allow the integration of smart solutions such as e-health, the Internet of Medical Things (IoMT), artificial intelligence, and machine learning for developing innovative healthcare technologies [16, 17]. Microfluidic technologies enable economic, fast, portable, and sensitive analysis opportunities, and offer versatility in development as the fabrication can be achieved with different material bases such as poly(dimethylsiloxane) (PDMS), poly(methyl methacrylate) (PMMA), polycarbonate, glass, paper, hydrogel, polytetrafluoroethylene (PTFE), thermoset materials, three-dimensional (3D) printing materials, and silicon [12, 18–20]. Microfluidic systems can be used for real-time sensing and monitoring, can work with small sample and reagent volumes, can allow multiplexing, and can be assembled into multiple microfluidic components [18, 19]. Therefore, those systems emerge as a great alternative to commercial detection and imaging systems.

The physical and chemical environment of the microfluidic systems can be precisely controlled, enabling a high-quality assessment that is required for viral biology research [21]. Translated to the clinic, early and accurate detection of viral diseases leads to early intervention, controlling the spread of disease and prompting clinical care by using microfluidic technologies [22, 23]. Microfluidic technology can offer superior capabilities for virus detection in terms of time, detection speed, and limits of detection [22]. Detection of the viruses can be conducted in either direct ways (i.e., an antigen, DNA/RNA are targeted via direct detection methods) or indirect ways (i.e., serologic tests that determine IgM and IgG antibodies in serum or plasma are used). Detection methods can further be improved by integrating them with artificial intelligence (AI) or internet-of-things (IOT) to perform point-of-care (POC) application during SARS-CoV-2 [24].

**Fig. 1:**
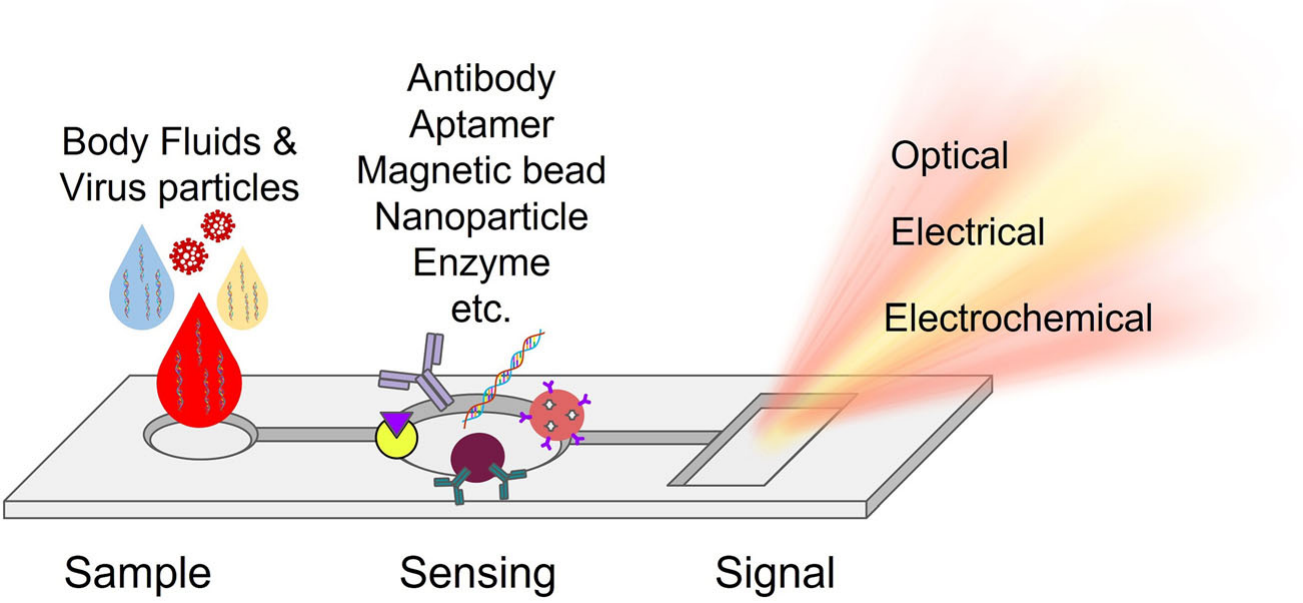
Overview of microfluidic methods used for detection of respiratory viruses. Various collected samples (genetic materials or direct respiratory virus particles in body fluids) can be detected using microfluidic-based detection systems that can contain antibodies, aptamers, magnetic beads, nanoparticles, or enzymes

Considering the increase of virus-based epidemics/pandemics and respiratory tract diseases due to these epidemics/pandemics, we aimed to compile current technologies that use microfluidic-based detection methods directly to the types of virus-related respiratory diseases. For this purpose, the types of viruses that cause respiratory diseases were given and conventional virus detection methods were explained. Moreover, the microfluidic-based direct detection methods used in the detection of these viruses in the last decade were explained in detail. The microfluidic-based direct virus detection systems (Fig. 1) are described based on the underlying detection methodology as optical, electrical, etc., and also commercial examples are also discussed. The importance of the use of microfluidics technologies in the detection of viruses that cause respiratory infections was emphasized. The difficulties encountered in virus detection were also explained. Hence, this review paper could be a handbook for researchers who will develop and use these microfluidic-based virus detection techniques.

## Respiratory Disease Viruses

Influenza viruses infect almost one billion people as acute respiratory disease and cause deaths over 500,000 in humans every year according to the WHO’s estimation [25]. Young children and elderly people who have chronic diseases are the most affected population subgroups [26]. Influenza viruses that are enveloped and have negative-strand RNA are categorized into three subtypes as influenza A, B, and C in humans [27]. Among them, influenza A and B are the most common types causing seasonal epidemics every year [28]. In the past, influenza A subtypes caused various pandemics. One of the deadliest influenza A pandemics was Spanish flu emerging in 1918. It is estimated that 500 million people were infected, and 40 million people died worldwide because of this pandemic [29, 30]. Asian flu (H2N2), Hongkong flu (H3N2), and Swine flu (H1N1) were the following influenza pandemics causing fewer mortalities [31]. On the other hand, the influenza B virus has not caused a pandemic before, however, it has caused local epidemic [32]. Symptoms such as fever, sore throat, headache, and nasal congestion are seen as indicators of influenza generally. The incubation time of the disease can change from 1 to 4 days. Rest and fluid intake are advised by physicians for healthy individuals for the treatment. Young children, elderly people, or people who have chronic health conditions may receive extra supportive medicine. There are available vaccines for the disease but, as virus strains mutate every year, vaccines are required to be updated yearly to remain effective in prevention [26].

Human adenoviruses (HAdVs), which are found in the *Adenoviridae* family, cause infections involving the respiratory tract, gastrointestinal tract, or conjunctiva, and pose a high danger to human health. HAdV is a non-enveloped and double-stranded DNA virus with 7 species and more than sixty serotypes [33]. Hemorrhagic cystitis, hepatitis, hemorrhagic colitis, pancreatitis, nephritis, fewer, or encephalitis are rare symptoms of HAdV infections. Adenovirus infections are more common in younger age groups due to humoral immunity deficiency. Besides, the severity of the disease, as well as its potential for transmission, is high in patients with impaired immunity. In closed or crowded environments, HAdV infections can reach epidemic dimensions, and their contagiousness increases [34]. HAdV, which has epidemic samples in certain regions, is a very suitable virus to reach pandemic sizes [35–38].

Human bocavirus (HBoV) is a non-enveloped, single-stranded DNA virus that belongs to *Parvoviridae* and mostly affects children by causing upper and lower respiratory infections [39, 40]. HBoV is the fourth most common pathogen found in respiratory tract diseases of children [41]. Other viruses have been found in HBoV-positive children with a rate of 37–90% [42].

The human metapneumovirus (hMPV), which belongs to the *Paramyxoviridae* family, is an enveloped and negative single-stranded RNA virus. It can cause severe respiratory disease in individuals with a low immune system [43–47]. Fever, cough, shortness of breath, bronchiolitis, and pneumonia are common symptoms in patients infected with hMPV [44, 48–51]. Although its recent discovery has accelerated the development of drugs and vaccines that effectively affect hMPV, methods are yet to be developed for the detection and treatment of this infection [47, 49].

The human rhinoviruses (HRVs) grouped into 3 different types as HRV-A, HRV-B, and HRV-C are positive single-stranded RNA virus within the *Picornaviridae* family [52–54]. These viruses, which preserve their genomic material in the capsid, interact with the intercellular adhesion molecule 1 (ICAM-1) receptors on the surface of the host cells [55]. HRV, which is a non-enveloped virus, can be seen in a wide range of patients from infants to the elderly, including patients with suppressed immune systems [52, 56]. Although HRV is known to be transmitted by the mucous fluid resembling a common cold, its infections can cause different respiratory diseases [57]. HRV, which brings on upper respiratory tract diseases, bronchiolitis, asthma, and chronic pneumonic diseases in the advanced stages of the infection, threatens human life because of fatalities [52, 58, 59].

Respiratory syncytial virus (RSV) is one of the enveloped, single-stranded, and negative-sense RNA viruses [60, 61]. It consists of two subtypes, types A and B, which are classified with their antigenic variability [62]. RSV is highly contagious and can infect people of all ages but predominantly observed among children and infants. It is one of the leading causes of illnesses such as bronchiolitis or pneumonia in young children [60, 61]. According to WHO, in 2017, over 30 million RSV-related infections in infants and children were estimated annually [63]. Also, elderly and immunocompromised people are reported as high-risk groups with significant morbidity and mortality [64–66]. RSV causes seasonal epidemics mostly beginning in the fall and peaking in the winter [60, 67]. In case of need, nasal/nasopharyngeal swabs or aspirations are mostly used to collect samples [68, 69]. Still, there is no effective vaccine against RSV; however, about 60 vaccine candidates are currently in different phase trials [63, 70].

Parainfluenza viruses (PIVs) are one of the enveloped single-stranded RNA viruses. They have 4 subtypes and are categorized in the *Paramyoviridaie* family. When infection occurs, viruses affect the upper and lower respiratory tract of infected people mainly. Symptoms start in the nose and then the virus can spread to lower airways in approximately 5 days. Parainfluenza viruses can infect any person worldwide and infection time can change according to seasons [71]. There is no vaccine or available antiviral treatment for these viruses, and only hygiene and sanitation practices are recommended [72].

Enterovirus 71 (EV71) is a member of the *Picornaviridae* family and is classified as a non-enveloped single-stranded RNA virus type. EV71 infects both children and infants but the infection is seen in children at a higher rate [73]. Infected children develop a disease called HFMD (hand, foot, and mouth disease) and China was the epicenter of the outbreak in 2012. General symptoms of the disease are fever and ulcers in the different areas of the body such as mouth and hands. Vaccines were developed in recent years for the prevention of the disease [74].

Coronaviruses (CoVs) are found in the *Coronaviridae* family and are classified into four groups as alpha, beta-, gamma-, and delta-coronaviruses [75, 76]. Coronaviruses are single-stranded RNA viruses that affect the respiratory system. CoV-229E, CoV-NL63, CoV-OC43, and CoV-HKU1 are four strains of human coronaviruses that show cold-like symptoms. Contrarily, severe acute respiratory syndrome coronavirus (SARS-CoV), Middle East respiratory syndrome–related coronavirus (MERS-CoV), and SARS-CoV-2 cause fatal lower respiratory diseases [4]. In 2003, SARS-CoV outbreaks in Asia infected more than 8000. In 2012, MERS-CoV was first seen in Saudi Arabia and, infected people showed severe acute respiratory symptoms such as fever and cough [77]. In late 2019, SARS-CoV-2 first appeared in China and rapidly became a worldwide pandemic [78]. As of 2020 December, there were about 65 million confirmed cases and over 1.5 million deaths [5]. SARS-CoV-2 is a crown-like enveloped single-stranded RNA virus that belongs to beta-coronavirus [79]. The early diagnosis of SARS-CoV-2 is important to prevent disease spread and the treatment [11, 80].

## Conventional virus detection methods

Many new methods have evolved or been invented to detect viruses. Medical professionals and researchers have faster, accurate, simple, and useful methods in the diagnosis, monitoring, and treatment of diseases caused by respiratory viruses. There are methods in which the findings are directly related to the virus such as nucleic acids, proteins, and virus particles. These methods are of great importance in virus detection by giving quantitative and/or qualitative results. Another method, indirect virus detection, is to detect the presence of the virus by investigating the metabolic changes (e.g., antibody amount) in the organism infected with the virus.

### Direct Detection

#### Polymerase Chain Reaction

Polymerase chain reaction (PCR) is a rapid, sensitive, and specific in vitro molecular technique that allows rapid amplification of specific segments of DNA, in most cases specific genes of interest [81]. The technique requires five main components: DNA template which contains the segment of interest of DNA to be amplified; two primers to determine the start and the end regions of amplification; Taq polymerase that copies the DNA amplified region; nucleotides which are used for making the new DNA with the DNA polymerase; and buffers for optimum DNA polymerase chemical environment [82]. Three main steps of PCR have repeated over 20–35 cycles which are denaturing of DNA, annealing of primers, and extension of DNA [83]. As a result, 100 billion of similar genetic materials can be produced via PCR.

The most common types of PCR are real-time PCR [84], reverse-transcriptase PCR [85], and multiplex PCR [86]. For RNA viruses, detection of viral RNA requires the synthesis of cDNA and amplification via PCR while for DNA viruses, DNA is amplified directly. Currently, PCR is the gold standard for respiratory infections, including COVID-19 diagnosis by identification of SARS-CoV-2-specific RNAs. Historically, PCR was used for the detection of several respiratory viruses. In 1998, Echavarria et al. introduced a new PCR method that amplifies the hexon gene to detect HAdV in urine in a fast and efficient manner [87]. The new assay was capable of detecting all 18 known stereotypes known at that time with optimized urine processing. In 2000, Xu et al. designed a PCR assay by using primers for fibers gene that in a single amplification reaction can differentiate HAdV from A to F types [88]. Besides, the assay could identify all 49 prototype strain species that were known at that time. In 2003, Gu et al. designed a multiplex PCR assay for several group of HAdV [89]. There are five primers that compose the assay and seven probes that can detect all adenovirus A, B, and E as well as 8 stereotypes from group D (stereotype G was not known at that time). In 2003, Heim et al. designed a quantitative PCR with primer and probe (TaqMan) sequences of the hexon gene for detection of HAdV [90]. Their designed model was capable of detecting all 51 HAdV prototypes. This method gave more sensitivity compared to the convenient PCR. In 2004, Ebner et al. developed a two-reaction real-time PCR assay covering all HAdV A to F with high specificity and sensitivity [91]. In 2008, Damen et al. designed a real-time PCR assay that can detect all known HAdV at that time [92]. They chose from the hexon region as well as degenerate primers to cover all known serotypes. In 2019, Dong et al. developed a method for rapid detection of fowl adenovirus serotype 4 (FAdV-4) and FAdV-10 in chicken through a droplet digital PCR (ddPCR) assay [93]. The authors assessed the qualification of the assay to detect FAdV-4 contamination in Newcastle disease virus vaccines and compared it to quantitative real-time PCR (qPCR) and a conventional PCR assay. In 2020, Nebeluk and Foster [94] published a method for the evaluation of adenovirus type 5 transcriptional patterns using a SYBR green-based quantitative qPCR. They developed a qPCR assay for the majority of the HAdV5 genome that allows the quantification of transcriptional activity. Through computational modeling, they used panel specific adenovirus gene primers that are compatible under a single reaction condition [94, 95].

In 2006, Lu et al. developed two real-time TaqMan PCR that is sensitive and effective towards HBoV [96]. They mainly targeted HBoV NS1 and NP-1 genes. Developed assays detect up to 10 copies of recombinant DNA plasmid with a HBoV genome’s partial region (10^1^ to 10^8^ copies). In 2007, Neske et al. developed another real-time PCR assay for the diagnosis of HBoV [97]. Their assay aimed to quantify the HBoV DNA and aimed to use it in the analysis of stool and serum samples for testing the presence of HBoV DNA. Their qualitative result corroborated with the conventional HBoV PCR. Their results were also important for confirming the presence of HBoV in stool. In 2008, Choi et al. described a novel method for the detection of HBoV based on real-time PCR assay [98]. They used the gene bank’s library to design primers and probes. For target sites, they analyzed the following genes as they were applicable for TaqMan real-Time PCR.

Other than HAdV and HBoV, major viral infections diagnosed with PCR are influenza, rhinovirus, syntactical virus, and enterovirus. In 1998, Echevarria et al. developed a method for the simultaneous detection of three serotypes of parainfluenza virus 1, 2, and 3 through RT-PCR multiplex assay [87]. The assay could detect and differentiate between the three types in a combined reaction. On conserved regions of the hemagglutinin-neuraminidase gene, a mixture of three pairs was used for primary amplification and yielding of amplicons of the same size. In 2004, Templeton et al. developed a method for detection of parainfluenza 1, 2, 3, and 4 as well as influenza A and influenza B viruses, and human RSV that uses molecular beacons to distinguish pathogens within a two-tube multiplex reaction [99]. In 1992, Claas et al. developed a method for influenza viruses A, B, and C by PCR [100]. In their method, they aimed to detect specifically for the RNA genome of each virus. Therefore, they chose three primers from the conserved regions of the genome coding for the non-structural proteins. In 2019, Henritizi et al. developed tetraplex RT-qPCR for influenza using primers and probes based on previously published assays [101]. In addition to that, more primers and probes for comprising full-length sequences were extracted from GenBank. The results proved that RT-qPCR is optimum, sensitive, and specific for the study of influence viruses and can be a powerful tool. In 2003, Mentel et al. developed a RT-PCR for syncytial virus as they used a primer/probe pair from the F gene [102]. This assay is specific for the detection of the virus and it has an advantage of the closed tube that eliminates contamination. In 2012, Anh Ha Do et al. developed a novel method for improved detection and quantification of syntactical virus [103]. Their method depends on quantitative RT-PCR that locks nucleic acid (LNA) probes to distinguish the groups A and B. In 1999, Andeweg et al. presented a method for the detection of rhinovirus using nested RT-PCR [104]. They designed primers and probes based on the most conserved regions to direct them there. In 2010, Do et al. developed a one-step real-time PCR for the detection of rhinoviruses [105]. They designed primers to amplify a target of picornavirus RNA with specific length and a TaqMan probes that were especially designed for specific detection of rhinovirus amplicon. This method allows sensitive detection of a variety of serotypes of rhinovirus with Applied Biosystems reagent-instrument platform. In 2008, Tan et al. presented a method for rapid detection of EV71 by real-time TaqMan RT-PCR [106]. They designed specific primers and probes based on theVP1 region of EV71. They proved that their method has 100% specificity to detect EV71 and sensitivity to 5 viral copies.

Regarding coronavirus studies, in 2004, Adachi et al. provided an RT-PCR assay that could detect SARS-CoV-1 and other members of coronaviruses (HCoV-OC43 and HCoV-229E) with high efficiency and analytical sensitivity [107]. They developed a single-tube RT-PCR. In the method, the species could be identified through sequencing amplicon. Also, in 2004, Emery et al. developed a real RT-PCR that could directly detect SARS-CoV-1 in a fast manner [108]. The idea of the assay is based on several probes and primers located in different regions of the genome of the virus that are capable of distinguishing SARS-CoV-1 from other coronaviruses with a LOD smaller than 10 genomic copies per reaction. In 2004, Wu et al. developed an RT-nested PCR system for the detection of SARS-CoV-1 [109]. In their study, they presented 12 sets of nested PCR that cover the entire genome sequence of SARS-CoV-1. The nested primers were screened, and they showed efficient sensitivity to detect the virus in RNA isolated from Vero 6 cells. The specificity was found to be 100% while sensitivity was found to be 83%. In 2012, Corman et al. presented two RT-PCR assays for SARS-CoV-1 [110]. They targeted the upstream region of E gene (upE) or the open reading frame (ORF) 1b, respectively. Their results indicated the sensitivity for the first one was 3.4 copies per reaction. They indicated the presence of no cross-reactivity with other SARS-CoV-1 viruses nor other coronaviruses OC43, NL63, and 229E. In 2013, Lu et al. developed real-time reverse transcription-PCR (rRT-PCR) assays for the detection of MERS [111]. In their method, the developed assays target MERS-CoV nucleocapsid (N) gene and they compared these assays with the ones targeting upE in MERS. In their results, they found that all the assays provided a detection rate of smaller than 10 copies/reaction of quantified RNA transcripts and 1.3 × 10^−3^ 50% tissue culture infective doses (TCID_50_)/mL of cell-cultured MERS-CoV.

In response to the current COVID-19 pandemic caused by SARS-CoV-2, in February 2020, Lu et al. developed a panel consisting of 3 RT-PCR assays to target the N gene in the virus [112]. They found that the limit of detection is 5 copies/reaction for all assays and 1 × 10^−1.5^ 50% tissue culture infectious dose/mL of cultured SARS-CoV-2. The assays were performed with serum, fecal specimens, nasopharyngeal, and oropharyngeal secretions spiked with the cultured virus. There was no detection for false-positive with other coronaviruses or respiratory pathogens. The US Food and Drug Administration (FDA) approved the panel for emergency use on February 4, 2020. In May 2020, Fuk-Woo Chan et al. developed and investigated the performance of three different RT-PCR assays that target RNA-dependent RNA polymerase (RdRp)/helicase (Hel), spike, and nucleocapsid genes of SARS-CoV-2 [113]. The lowest limit of detection in vitro was the COVID-19-RdRp/Hel assay with 1.8 TCID_50_/mL with genomic RNA and 11.2 RNA copies/reaction with RNA transcripts. The assay did not show cross-reacting activities with other respiratory pathogens or other coronaviruses compared to RdRp-P2 assay which cross-reacted with SARS-CoV-1. The assays showed to be highly sensitive and can detect cases that were not detected by RdRp-P2 assay.

#### Loop-mediated isothermal amplification

A new milestone in the field of molecular biology was through the use of thermostable DNA polymer in PCR as primers [114]. Nevertheless, due to the limitations of PCR in general such as the need for complex instruments, other more practical methods were desired. Several isothermal techniques were developed at the beginning of the new century. Loop-mediated isothermal amplification (LAMP) technique was developed and introduced for the first time by Tsugunori Notomi and his colleagues in 2000 [115]. The technique is unique as it is capable of amplifying DNA efficiently with a one-step reaction [116]. LAMP relies on two components: DNA polymerase, and four designed primers that are capable of the recognition of six distinct regions of the DNA [115] (i.e., two of these regions are outer primers and the others are inter primers) [ 117]. The process in LAMP consists of non-cyclic and cyclic steps. In the non-cyclic steps, artificial stem-loops are generated to be used in the cyclic steps. In the first cyclic step, the FIP binds to the sequence of targets to initiate polymerization. Consequently, F3 binds to the product displacing it with a stem-loop adding to the targeted sequence. Then, the DNA single-strand behaves as a template and the B3 binds to the product and displaces it with two adjoining artificial stem-loops. The second cyclic step then takes a place with hybridization of FIP to the loop on the product. Initiation of DNA placement synthesis then takes place producing DNA with a new stem-loop structure and an additional target sequence. For further DNA synthesis, these structures will be used as templates in addition to internal primers. Inverted repeats of target sequence are obtained from different structures [118].

Upon its introduction, LAMP has been used for the identification of different respiratory viruses, including influenza, rhinovirus, enterovirus, and coronavirus. In 2003, Wakabayashi et al. presented a method for fast and sensitive diagnosis of adenoviral keratoconjunctivitis by LAMP [119]. In their method, they used an adenovirus (ad) type-specific primer for the gene of ad1, ad3, ad4, ad8, ad19, and ad37. In the study, the authors showed that the genotype of LAMP samples was identical to the PCR. In 2014, Sun et al. compared different DNA extraction methods, boiling, boiling in 1% Triton X-100, and treating 0.02 M NaOH with DNAzol DNA extraction method [120]. Compared to the DNAZol method, the specificity of all three was 100% for adenovirus with the boiling method the most sensitive. The extracted template from supernatants of nasopharyngeal aspirates was further analyzed and showed higher sensitivity and specificity in LAMP assay compared with those for PCR. In 2006, Ito et al. developed RT-LAMP assays for the detection of influenza A virus H1 and H3 subtype strains and influenza B virus strains specifically [121]. For specificity, the strains were detected by strain-specific primers. For sensitivity, the virus was detected with a minimum concentration of 10 ffu/m. The assay was shown to be more sensitive than immunochromatography and was found to be useful for diagnosing emerging influenza subtypes. In 2019, Nakauchi et al. developed two assays for the detection of rhinovirus by real-time fluorescent RT-LAMP [122]. The first assay was designed based on the 5′-untranslated regions (UTRs) of rhinovirus A and B and the second assay was designed based on the 5′-UTR of rhinovirus C. Efficiency of both assays was tested and compared with RT-PCR. Their sensitivity for rhinovirus A was 86.3% and for rhinovirus C was 77.3% on clinical specimens by the combined use of both assays. No cross-reactions were reported. In 2013, Mahony et al. developed a rapid and sensitive multiplex LAMP assay for the detection of respiratory syncytial virus subgroups A and B (RSV A and B) [123]. In their study, primers were designed for RSV A matrix gene and RSV B polymerase gene. Their assay had a 100% sensitivity and specificity. Their total diagnosis time was 30 min. In 2014, Mu et al. developed RT-LAMP for 1-h detection for simultaneous detection of A and B groups of human syncytial virus [124]. They designed primers for groups A and B that specifically amplify the N and L genes of the virus. The limit of detection of new method was 281.17 and 1.58 TCID_50_/mL for HRSV A and hRSV B, respectively. The test was useful for rapid detection. In 2019, Hu et al. developed an RT-LAMP method for simultaneous high detection of hRSV A and hRSV B group [125]. The primer designed for A group was on M gene while the primer designed for B group was on M2-2 gene. For the amplification of hRSV RNA, real-time monitoring was achieved using SYTO9 as the fluorescent dye. In comparison of the new LAMP method with conventional RT-PCR, the positivity rate of RT-LAMP and RT-PCR was found as 67.8% and 55.6%, respectively. In 2011, Wang et al. developed a single-step RT-LAMP for the detection of enterovirus 71 [126]. The assay takes place in one tube and approximately 1.5 h at 65 °C. They found that the detection limit was about 10 copies. In addition, they found no cross-reactions with coxsackievirus A16, echovirus, HRV, or norovirus. Compared to conventional RT-PCR, this assay had greater sensitivity. In 2011, Yaqing et al. developed a one-step single-tube RT-LAMP for the detection of human EV71 [127]. The assay targeted the amplification of the VP1 gene in the presence of specific primers kept with DNA polymerase for 1 h at isothermal temperature conditions of 63 °C. The product was assessed through visual inspection and agarose gel-electrophoresis. After RNA extraction, compared to conventional RT-PCR, it was found that the assay was 10-fold more sensitive. The assay was very specific and showed no cross-reactivity.

Regarding coronavirus studies, in 2003, Thai et al. presented a new assay called a one-step single-tube accelerated RT-LAMP assay for SARS-CoV-1 detection for replicate gene [128]. Compared to conventional RT-PCR, their RT-LAMP was 100-fold more sensitive with a 0.01 PFU detection limit. The sensitivity and specificity of RT-LAMP compared to RT-PCR were 100 and 87%, respectively. The amplification in RT-LAMP was carried out at 63 °C in a single tube under isothermal conditions. In 2017, Hee Lee et al. developed a one-Pot RT-LAMP assay for detecting MERS-CoV [129]. In their method, they designed six LAMP primers using the sequence of nucleocapsid (N) gene: 100 U M-MLVRTase and 4 Bst Polymerase. This means that the reaction has the ability to detect four viral copies in 60 min. They used EvaGreen dye instead of SYBR green because it gives better signal readout properties in one-pot RT-LAMP reaction as well as it has excellent binding properties with DNA polymerase. In 2015, Bhadra et al. developed a RT Sequence-LAMP Assays for detection MERS-CoV [130]. Their method consists of isothermal amplification assays for MERS-CoV using open reading frame (ORF)1a and ORF1b genes and upstream of E gene (upE). In each assay, an incorporation of a single loop primer took place, and it affected the asymmetric amplification leading to an acceleration of the time-to-result of the OSD-RT-LAMP assay. The assays have a detection ability of 0.02 to 0.2 plaque-forming units (PFU) (5 to 50 PFU/mL) of MERS-CoV between 30 and 50 min. There was no cross-reaction. In 2018, Huang et al. provided a mixed technique that utilized rRT-LAMP, and vertical flow visualization strip (RT-LAMP-VF) for sole purpose of detection for N gene in MERS-CoV [131]. This assay could be performed in a constant temperature for about 30 min and a colorimetric result could be visible to the naked eyes within 5 min. The technique could detect synthesized RNA transcript and MERS-CoV RNA at 2×10^1^ copies/μL and 1×10^1^ copies/μL. There was no presence for cross-reactivities, and the method was highly specific.

In the early months of COVID-19 pandemics, Huang et al. applied RT-LAMP to achieve detection in 30 min for SARS-CoV-2 detection [132]. In their assay, they designed four sets of LAMP primers and each set contained 6 primers to target the viral RNA of the virus in the 1ab, S gene, and N gene regions. They produced a colorimetric response that is capable of naked-eye viral RNA detection. The reaction is a one-step process that does not require RNA extraction. The sensitivity was as high as 80 copies per milliliter of viral RNA. The results were in agreement with conventional RT-qPCR. Park et al. developed an RT-LAMP assay for genomic RNA of SARS-CoV-2 [133]. Their assay could detect 100 copies per milliliter of SARS-CoV-2 RNA. There were no cross reactions with other respiratory pathogens nor coronaviruses. The assay adapts a colorimetric detection for high throughput. Hu et al. developed a novel RT-LAMP assay for SARS-CoV-2 and compared them to RT-qPCR [134]. They found that the RT-LAMP had 88.89% sensitivity and 99.00% specificity. Compared to conventional RT-qPCR with 81.48%, they had an improved sensitivity of 88.89%. Furthermore, no cross reactions were detected with other respiratory pathogens or coronaviruses.

#### Microarray

Microarray is another fascinating direct molecular technique that was established in 1990 by Patrick Brown and his team [135]. A spot or feature in a microarray is the area where a specific probe is located. In the solid support, the probes are immobilized and the targets are applied as a solution onto the array for hybridization after fluorescent labeling [135, 136]. The DNA microarray possesses the size of a fingernail and it has at least 96 wells, each of them containing thousands of oligonucleotides or probes that are arranged in a grid manner [136–138]. There are two types of microarrays: cDNA (made by using robotic pins to print double-stranded cDNA on a solid support) and oligonucleotide (made by using photolithography to synthesize specific oligonucleotides in a specific alignment on a solid surface) [136, 139]. Hybridization can take place after the labeled cDNA is applied to microarray. Following the slide wash, it is expected that nonspecific hybridization is removed, and it is then read in a laser scanner that has the ability to differentiate between Cy3 and Cy5 signals allowing the production of separate 6-bit TIFF image for each channel due to the fluorescence intensity collected. cDNA pools that are reverse transcribed from mRNA samples can be distinguished with Cy3 and Cy5 fluorescent dyes. Quantification of fluorescent intensity is corresponding to gene expression in a sample. Since its discovery, it has been applied for efficient, specific, and sensitive amplification of both DNA and RNA from a variety of organisms and biological compartments [116]. A typical microarray consists of pieces of DNA that range from 20 to 5000 base pairs embedded into a designated area on a solid support. Furthermore, protein microarrays can also be used for the detection of protein-based biomarkers for diagnostic test [140, 141]. In these arrays, antibodies are extensively used as probes [142–144]. Adaptation of microarray to respiratory virus detection is fairly new. In 2003, Shih et al. developed a microchip for detection of enterovirus. In their approach, the amplified DNA was hybridized with oligonucleotide immobilized on a microchip [145]. For probes, two oligonucleotides were used: 5′-noncoding region (5′-NCR) sequence of the pan-enterovirus and the enterovirus 71–specific sequence of VP2 region. The specificity was found to be 90.0% while sensitivity was 89.6%. The microchip array for enterovirus 71 could detect the amplicon of viral RNA as 1-10 virions in the specimen. In 2007, Quan et al. established and validated method for a sensitive microarray system for the detection of influenza viruses [146]. They were able to accurately characterize twenty one respiratory viruses. In 2007, López-Campos et al. developed a microarray assay for the detection of adenovirus [147]. They utilized amplicon retrieval software and positive controls as reference strains serotypes 1, 2, 3, 4, 5, 7, 14, and 21. Other strains were used for control of specificity. To assess sensitivity and specificity, additional controls of cloned amplified adenovirus type 1, influenza virus (A, B, C), and respiratory syncytial virus (RSV A and B) were used. They successfully managed to develop an oligonucleotide microarray that could identify and detect adenovirus serotypes that are linked with respiratory infections accurately and efficiently. In 2018, Nybond et al. developed a microarray system composed of isothermal amplification of viral DNA with a paper-based vertical flow microarray (VFM) using functionalized gold nanoparticles (AuNPs) for colorimetric detection of amplicons [148]. They tested an in-house-designed probe and an adenoviral probe to validate microarray detection using synthetic target DNA down to 50 nM. In a recombinase polymerase amplification, the primers were proven to function using the synthetic template and viral DNA. The authors demonstrated the detection of adenovirus with four adenoviral species using the paper-based VFM. The assay could detect intra- and inter-assay CV% of ≤ 9% and ≤ 13% from 1 ng of starting material.

In 2004, Long et al. developed a universal microarray that integrates RT-PCR and ligase detection reaction (LDR) for the detection of SARS-CoV-1 [149]. For creating the universal microarray, the zip code attached to a side remaining constant and their complementary cZip codes were used for tagging the target sequence. The 5′end “cZip Codes” directs the product of LDR to specific codes linked covalently to a slide. In 2014, Guo et al. developed a microarray for the detection and genotyping of SARS-CoV based on a single-nucleotide polymorphism (SNP) target [150]. PCR was used for the hybridization of the product amplified from cDNA synthesis from different strains of SARS-CoV. The authors were able to detect 24 SNPs and determine their strain types. The hybridization was detected and genotyped with 100% accuracy using the microarray.

#### ELISA

Enzyme-linked immunosorbent assay (ELISA) is a molecular detection method that is based on the enzyme-labeled immunoassay [151]. One of the works that paved the way for the discovery of the method is the work of Aarameas that demonstrated the successfulness of the coupling of antigen-antibody through the use of several enzymes [152, 153]. Two scientists named van Weemen and Schuurs described it independently as well in the same year in their paper entitled “Immunoassay using antigen-enzyme conjugates” [154]. In their work, they conjugated the antigens from human chorionic gonadotropin (HCG) to the enzyme horseradish peroxidase (HRP) and they used purified conjugates for “enzyme-immunoassay” of antibody and antigen. In general, an ELISA system takes advantage of enzymes that are attached to one reactant in the immunoassay followed by the addition of a proper substrate or chromogen that allows a colorimetric response to take place. The most common ELISA type is the solid-phase heterogeneous ELISA. For solid-phase heterogeneous ELISA, there are four main types: direct ELISA, indirect ELISA, sandwich ELISA, and competitive ELISA. Through the washing step, flooding and emptying the well using buffered solution ensures for successful separation of bound (reacted) from unbound (unreacted) reagents in the ELISA. Finally, the color development system allows results to be read and obtained through a spectrophotometer [155].

In 1979, Hamron et al. developed a solid-phase ELISA antihexon serum which was used for the detection of adenovirus antigen in cells [156]. Their results showed that in HEp-2 cell cultures, with 10 (2.5) TCID_50_, antigens could be inoculated and 10 (1.5) TCID_50_ after 2 and 4 days of incubation. Following 2 days of incubation, ELISA positive rate was 62% and no cytopathic effect was observed. After 4 days, ELISA positive rate was 76% and the cytopathic effect was 47%. The immunofluorescent method and ELISA were nearly identical in results. In 1989, Al-Nakeb et al. developed a novel ELISA from nasal washings for direct detection of rhinovirus [157]. The new ELISA detected infection in volunteers indicating infection in a higher proportion of asymptomatic volunteers than the symptomatic ones. In 2014, Zhan et al. developed an assay for the detection of syncytial virus [158]. In their method, gold nanoparticles were used for the detection of the virus as carriers of the signaling antibody anti-RSV-HRP to achieve amplification of the signal. The advantage of this assay compared to conventional assay for the same virus was that it achieved a shorter time and higher sensitivity between 0.5 and 50 pg/mL. In 2016, Leirs et al. developed a digital ELISA for detecting influenza A [159]. Seven commercial antibodies were selected to target influenza’s nucleoproteins. There were two different platforms in that study: ELISA and surface plasmon resonance system. The antibodies behaved differently in each platform but overall they achieved good reactivity in both.

Regarding coronavirus work, in 2004, Lau et al. developed an ELISA for detecting SARS-CoV-1 nucleocapsid protein [160]. They utilized hyperimmune polyclonal nucleocapsid-specific antibodies and SARS-CoV-1 nucleocapsid protein with His6-tag. It was aimed to detect nucleocapsid protein of the virus in nasopharyngeal aspirate, urine, and fecal samples of infected patients that were collected between 2 days and 33 after confirmation of the infection. The specificity in hospitalized patients without SARS for nasopharyngeal aspirate was 96.7%, urine is 99%, and the fecal specimen was 96%. As for SARS patients, assay detection was 34 (52%) of nasopharyngeal aspirate samples, 5 (5%) of urine samples, and 36 (55%) of fecal samples. For SARS-CoV-2 detection, in October 2020, Schöler et al. developed a novel In-Cell ELISA (icELISA) assay for automated detection in 48 h [161]. They employed this approach suitable for direct antigen source for quantitative icELISA from SARS-CoV-2-infected cells. The specific signal of SARS-CoV-2 reduced depending on antiviral interferons and human sera containing virus-neutralizing antibodies (NAbs). Upon the application of increasing infectious doses, the icELISA-based neutralization test (icNT) was superior to plaque reduction neutralization tests (PRNTs) in the differentiation of convalescent sera with high sensitivity from others. Furthermore, they found that icNT is specific, in differentiating between SARS-CoV-2-specific NAbs and those triggered from other coronaviruses.

#### Aptamer-based detection

Aptamers are defined as artificial nucleic acids composed of single-stranded DNA or RNA, but can also be defined as a combination of unnatural nucleotides that act similarly to ligands that coordinate 3D folding of these proteins [162]. Aptamers are classified by loops, hairpins, pseudoknots, bulges, triplexes, and/or quadruplexes [163]. Aptamers can be useful in several applications such as generations of enzyme inhibitors, analysis of nucleic acid recognition, analysis of hormones and toxins, detection for the presence of target molecules, and generation of lead compounds in medicinal chemistry [163]. They can be described as relatively new technology and they have not been applied to many practices. Nevertheless, a few interesting innovations have been explored.

In 2008, through the use of exponential enrichment (SELEX) for H5N1 influenza virus, the selective evolution of ligands that screen DNA aptamers targeting recombinant HA1 proteins was utilized by Cheng et al. [164]. Eleven rounds of selection were performed and 10 aptamers were found with a strong binding to HA1 protein and an inhibitory impact to the H5N1 virus in hemagglutinin and MTT assays. In 2013, Wang et al. presented aptamers for the H5N1 virus based on SELEX and surface plasmon resonance (SPR) [165]. They selected aptamers after 13 rounds. They showed a strong affinity in terms of binding between HA and the chosen aptamer. They showed negligible cross-reactivity with other non-targets such as H5N2, H5N3, H5N9, H9N2, and H7N2. The aptamer showed promised selectivity towards H5N1. In 2013, an aptamer was developed by Shiratori et al. for multiplex influenza A virus subtypes that can bind to the HA1 protein using SELEX [166]. Besides, they developed a sandwich detection method based on aptamer to employ new determined aptamers. The developed enzyme-linked aptamer assay system was able to detect influenza A subtypes that were H5N1, H1N1, and H3N2 with equal sensitivity. In 2006, Gopinath et al. improved an aptamer-based method for sensing of influenza virus B [167]. In their method, they isolated RNA aptamer through an in vitro method and this aptamer was found to be efficiently bound to the HA of influenza B and involved 5 mM of MgCl_2_ ion for its identification. In their findings, the aptamers can differentiate between influenza A and B. In 2014, Lai et al. developed an integrated microfluidic SELEX system to determine aptamers for influenza A/H1N1 (InfA/H1N1) virus in an automated mode [168]. In magnetic bead assay, the selected aptamer demonstrated high specificity and sensitivity detection towards InfA/H1N1 virus, even in biological samples such as throat swabs. Besides, 20 aptamers showed outstanding affinity for InfA/H1N1. In 2017, Percze et al. provided a method that relied on the use of aptamers for sensing of syntactical viruses [169]. In their method, the SELEX protocol was followed to select aptamers. The aptamers were generated through a single molecule as the target of selection. The aptamers showed high selectivity towards syntactical viruses.

In July 2020, Zhang et al. reported the first DNA aptamer for targeting nucleocapsid protein of SARS-CoV-2 [170]. They were able to obtain four DNA sequences with an affinity of down to 5 nM after five rounds of selection. The best binding towards the protein was with 0.49 nM Kd value. The four aptamers could bind successively to the protein in what they believe that it is a sandwich-structured interaction. The protein at the tens of pM level was successfully detected using ELISA and immunochromatographic strips. In October 2020, Liu et al. described a sensor for COVID-19 diagnosis using aptamers [171]. In the sensor, two aptamers could probe to the identical protein target and pull the ligation DNA field, thus initiating ligation-confirmed qPCR amplification. In their method, they were able to detect serum nucleocapsid quantitatively through the conversion of protein recognition into a detectable qPCR signal. The system was utilized and became a detection platform for special interactions between Spike S1 and the ACE2 receptor.

### Indirect Detection

Although direct virus detection based on specific antigen or nucleic acid is preferable, there is also an indirect detection methodology based on the assessment of antibodies generated by the patient as a reaction to infection. This serological detection technique is widely used in research and clinics; however, it has disadvantages compared to direct detection, such as low accuracy and low specificity [172].

Immunoglobulin M (IgM) and immunoglobulin G (IgG) are antibodies created by the human body after infection [173]. Serum levels of both antibodies are indicators for infections. While IgM antibody is more related to the early stage of the infection, IgG antibody is used as an indicator of the middle or late period of the infection. So, combinations of these two antibodies are used for sensing of viral infections or determination of stages of the infection [173]. When the literature examples are examined related to respiratory virus detection through indirect methods, SARS-CoV-2 diagnosis was made by using a combination of IgM and IgG antibody detection [173]. Method used for the experiments was chemiluminescence immunoassay and experiments were carried out successfully for patients. According to results, assessment of IgM and IgG antibody combination showed better sensitivity and that would be a method to detect SARS-CoV-2. As a different example, detection of SARS-CoV-2 was aimed by using IgM and IgG antibodies and levels of antibodies were determined through chemiluminescence immunoassay [174]. So, this method was offered for diagnosis of COVID-19 disease. In another work, investigation of the potential of rapid IgG/IgM test by lateral flow assay (LFA) was conducted for SARS-CoV-2 and compared with ELISA results [175]. Two main results of the study were that a combination of IgG and IgM antibodies did not increase specificity of the detection potential and that only IgG antibody detection without IgM antibody by LFA is more specific than by ELISA. Enzyme immunoassays were used to detect parainfluenza IgG and IgM antibodies [176]. When the levels of antibodies were measured quantitatively, it was shown that IgG antibodies reached more numerical value as percentage in patients infected by parainfluenza viruses.

Roggendorf et al. developed an ELISA assay by subjecting antibodies to hexone antigen of adenovirus through antigen-coated microtiter plate and peroxidase-coupled anti-human IgG followed by the addition of orthophenylenediamine and measuring the absorbance [177]. The authors discovered that the ELISA was 100-fold more sensitive than complement fixation. Barclay and Al-Nakib developed an ELISA for sensing of specific antibodies of rhinovirus in human sera and nasal secretions [178]. Rabbit anti-rhinovirus hyperimmune serum was used as the capture antibody via ELISA method. This ELISA system was proven to sensitively detect the rhinovirus-specific antibody for IgG and IgA immunoglobulins in serum. Wang et al. improved a detection method for EV71 through ELISA [179]. In their method, they relied on enterovirus 71-IgM-capture ELISA. The sensitivity and specificity of the assay were found as 97.7 and 93.3%, respectively. MacMullan et al. published a method for SARS-CoV-2 detection in saliva through ELISA [180]. In their method, commercially available Gold Standard Diagnostics (GSD) and EuroImmun (EI) kits were used to detect nucleocapsid protein (N) and spike protein (S), which are SARS-CoV-2 structural proteins to efficiently detect IgA and IgG antibodies. Both IgG and IgA kits from GSD and IgG kit from EI were found as 100% specific, while IgA kit from the EI was found as 92% specific for serum samples. They chose the EI IgG kit for the optimization and saliva experiment. Two different methods were compared for saliva collection: using oral fluid specimen collection device from OraSure Technologies and a mouthwash prepared from an in-house formulation using a subset of saliva samples. They found that the mouthwash yielded 100% sensitivity while the OraSure Collection Device yielded only 87% sensitivity. Furthermore, they managed to achieve a total 84.2% sensitivity and 100% specificity in a set of 149 clinical samples.

## Virus detection in microfluidic devices

### Optical detection techniques

In this section, we reviewed recent studies that make original and innovative contributions to the literature about optical detection of respiratory virus including absorbance, surface plasmon resonance, localized surface plasmon resonance, fluorescence, naked-eye or colorimetric, and others in microfluidic devices. Methods were explained by referring to their contributions for the virus detection, used labels, limit of detection (LOD), and detection methodology in detail.

#### Absorbance

Absorption spectroscopy technique is an important factor in molecule detection and laboratory diagnostics. In this technique, the attenuation or intensification of the wavelength of the light is measured with a spectrophotometer. The spectrum obtained in the spectrophotometer is measured as absorption differences that help define the concentration or composition of the molecule or sample to be determined [181, 182].

**Fig. 2:**
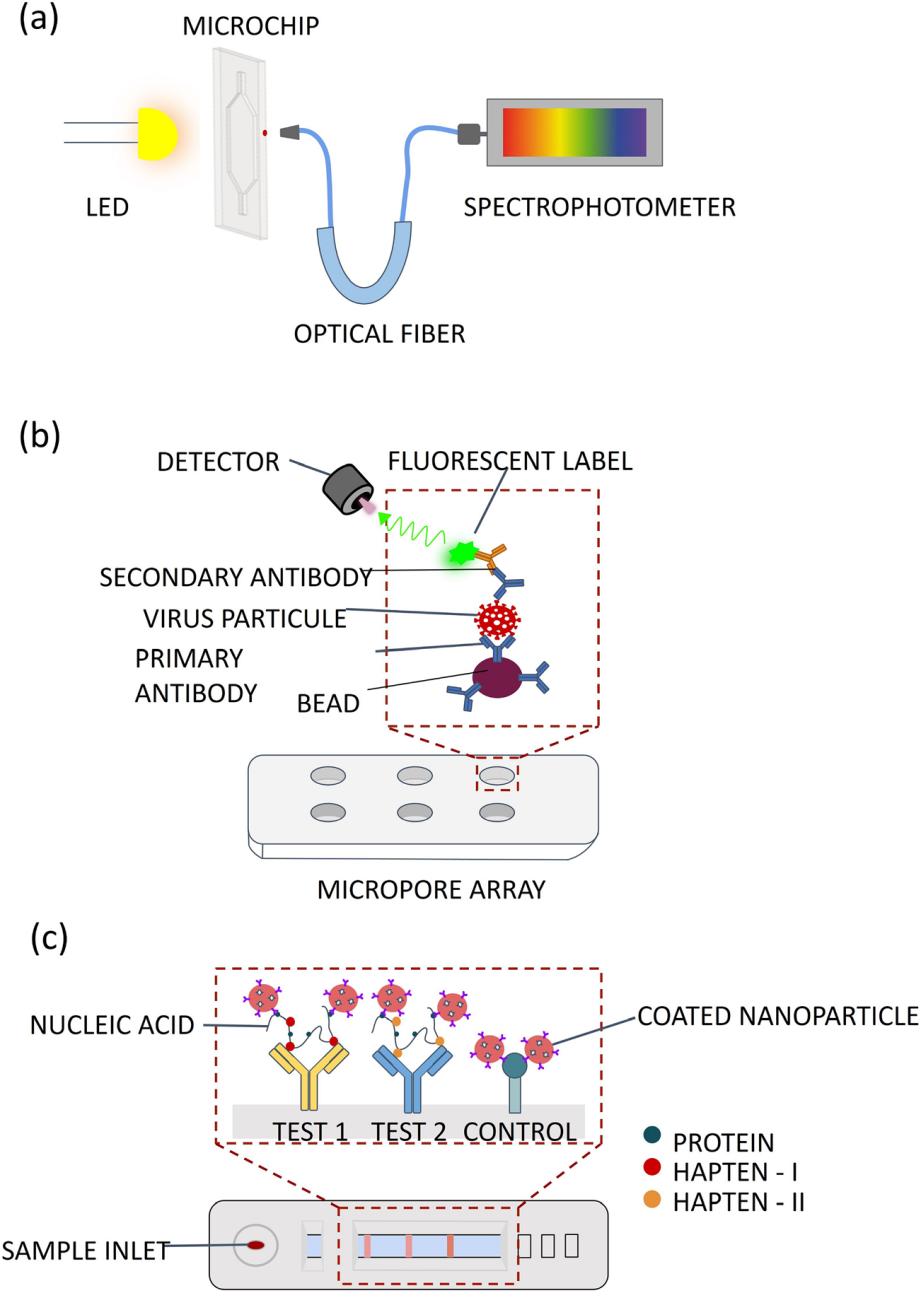
Illustrations of optical-based microfluidic platforms for detection of respiratory viruses or their products. **a** Absorbance-based detection technique, **b** Micropore array used for fluorescent detection. **c** Colorimetric detection in a paper-based microfluidic platform using RT-LAMP

With the evanescent wave absorbance technique, the light-emitting diode (LED) is connected to the optical fibers and measured with a suitable photodetector at the output. In this method, a local change in the refractive index affects the absorbance with an analyte-originator, and the change in absorbance is measured (Fig. 2a). This method has been used for molecular detection such as immune sensitive biosensing, detection of unicellular organisms such as bacteria [183], and analysis of heavy metals [184], proteins [185], antibiotics [186], and biological biomarkers, and it has also been shown that it can be used for direct and indirect detection of the SARS-CoV-2 virus [187]. Using the plasmonic fiber-optic absorbance biosensor technique, direct detection of SARS-CoV-2 virus particles without washing was achieved by measuring the loss of optical power (absorbance/ intensity change) in light [188]. In the designed method, binding of SARS-CoV-2 or free N-protein to capture antibodies attached to the surface provides a decrease in light intensity. The most important feature of the method is that the results can be obtained in around 15 min without performing sample preparation. Moreover, the influenza virus can be detected with grayscale images using an absorbance-based method [189]. It enables the detection of influenza virus strains by coating of polydopamine/protein G mixture and immobilization of an antibody against pH1N1. Absorbance-based methods can be used for direct detection of viral nucleic acids, such as influenza A, SARS-CoV-1/2, using LAMP with integrated optical fibers into a chip [190].

#### Surface plasmon resonance

SPR sensors are used for highly sensitive and real-time detection. SPR is defined as the condition of surface plasmon as a result of the interaction with a photon on a metal-dielectric interface [191, 192]. SPR sensors make analyte detection by measuring refractive index changes of the surface. They have wide range applications in environmental and medical diagnostics based on detection of target analytes such as proteins, nucleic acids, and viruses. Properties of SPR sensors providing label-free and sensitive detection make them essential tools [191–195]. Localized surface plasmon resonance (LSPR) sensors developed by nanostructured substrates are improved versions of SPR sensors. Usage of nanostructures in LSPR sensors provide reliable, faster, more responsive and sensitive detection of analytes when compared to SPR sensors [191, 196, 197].

SPR biosensor was used for detection of 9 respiratory viruses which are influenza (A and B), respiratory syncytial virus (RSV), parainfluenza virus (1–3), adenovirus, and severe acute SARS within 30 min [74, 198]. Nine oligonucleotides specific to respiratory viruses were immobilized on gold SPR chip. Labeling of oligonucleotides was made by synthetic amino (NH_2_) groups. SPR biosensor technology was used for avian influenza A (H5N1) antibody biomarker detection with an assay time of 60 min [199]. SPR sensor increased the detection limit (193.3 ng mL^−1^), which is more than 3-fold compared to available commercial systems for H5N1 antibody biomarker. A label-free SPR method was used for human EV71 detection [200]. This miniaturized and portable platform was built by attachment of color tunable organic light-emitting diode to prism. As a biomarker, a major capsid protein of EV71 (VP1) was selected. Detection limit of this highly sensitive SPR-based sensor was about 4.8 pg/mL. In another study, plasmonic biosensor combined with LSPR technology was used for SARS-CoV-2 detection [32, 201]. Combination of gold nanoislands (AuNIs) and complementary DNA receptors was made for SARS-CoV-2 detection through hybridization. On the AuNIs chip, the thermoplasmonic heat was generated to enhance sensing abilities. Developed label-free LSPR biosensor showed high sensitivity and low detection limit (0.22 pM) for SARS-CoV-2 detection from multigene mixture. In a different study, a magnetic and reusable SPR sensor chip was designed and used in conventional SPR systems for testing detection of H1N1 nucleoprotein detection [202]. In the sensing step, ferromagnetic patterns were selected to trap magnetic particles on SPR surface. Immobilization of antibodies was also made by EDC-NHS coupling method onto magnetic particles. After sensing step, magnetic particles held by ferromagnetic patterns separated from the chip by using external magnetic fields to make the SPR sensor chip reusable with new magnetic particles.

#### Fluorescence

Fluorescence detection method is commonly used in nucleic acid detection by using labeled fluorescent reporters to detect targets. Currently, 80% of the SARS-CoV-2 detection methods are performed by fluorescent signal detection of PCR products [11].

Electrical field combined microfluidic CRISPR–based detection provides 30 min assay time for the SARS-CoV-2 detection from the raw sample [203]. In this system, CRISPR-Cas12 enzyme and synthetic guide RNA (gRNA) was complexed and this complex specifically bound to target DNA. Viral N and E genes and human RNase P genes were targeted. Then, fluorophore-quencher-labeled ssDNA reporter probes was cleaved by this complex and fluorescent signal was observed. Electrical field was used to control and accelerate the CRISPR assay. Isotachophoresis (ITP) can separate charged analytes based on their ionic mobility by applying an electrical field. Thus, the extraction and automated purification of target was achieved. LOD of this system was found as 10 copies/μL with a consumption of less than 0.2 μL reagent. In this platform, the electrical field addressed the challenges of conventional CRISPR applications. This study is really advantageous due to no requirement for the separate nucleic acid extraction and on-chip extraction only takes 5 min. Simultaneous detection of influenza viruses (H1N1, H3N2, H9N2) was achieved by nucleic acid hybridization with controllable micro-magnetic field to create a magnetic reaction area for cDNA recognition by capturing magnetic nanoparticles modified with capture probe DNAs (CP-DNAs) on a microfluidic chip platform [204]. Quantum dot (QD)–assisted fluorescence signal measurement was achieved with LOD of 0.21 nM for H1N1, 0.16 nM for H3N2, and 0.12 nM for H9N2. In this system, the sample and reagent consumptions were only 3 μL.

A developed microfluidic system allowed detection of multiple influenza viruses, such as influenza A H1N1, H3N2, and influenza B, in 20 min [205]. In this study, a single universal aptamer was used due to its ability to recognize influenza viruses and also ability to change conformation depending on the different conditions, so different influenza viruses could be identified. After the mixing of universal aptamer-coated magnetic beads and viral sample, fluorescent-labeled single universal aptamer was added to the reaction chamber. LOD of this system was found to be 3.2 hemagglutinating units (HAUs), significantly lower compared to conventional hemagglutinin assays that have a typical LOD of 32 HAU. Immunomagnetic bead-based microfluidic system was designed for the detection of influenza A virus [206]. Viral particles bound to immunomagnetic beads and optical signals of magnetic complexes were analyzed. Integrated suction-type microfluidic control module, incubation module, and optical detection module performed sample incubation, purification, and optical detection automatically. Influenza A viral particles in the sample were captured by mouse anti-influenza nucleoprotein (NP) monoclonal antibody (mAb)–conjugated magnetic beads. Then, another mouse anti-influenza NP mAb labeled with R-phycoerythrin (PE) was incubated on magnetic beads. Finally, fluorescent signals were detected by an integrated optical detection module (Fig. 2b). LOD was found as 5×10^−4^ HAU which is much better than conventional bench-top systems. Another study was performed for the multiple virus detection using fluorescence magnetic multifunctional nanospheres [207]. Fluorescent magnetic multifunctional nanospheres were prepared by combining magnetic nanoparticles and quantum dots with different emission wavelengths. Green, yellow, and red fluorescent magnetic nanospheres (GMNs, YMNs, RMNs) were conjugated with antibodies against H9N2, H1N1, and H7N9 avian influenza viruses (AIVs). In antibody-modified micropore arrays, fluorescent images of micropores were observed after sandwich immunoreaction. According to their result, each fluorescent nanosphere on micropores shows single virus detection ability. When three viruses were present in the sample, these viruses can be observed simultaneously according to their fluorescence signal with a LOD of 0.02 pg/mL.

#### Colorimetric

Colorimetric detection techniques, which offer the possibility of detecting with a naked eye, can be highly preferred because of their low-cost and rapid measurement features. Direct and sensitive detection can be performed with these systems based on the reactions or aggregation of nanoparticles [208].

Detection of influenza A H1N1 and H3N2 from cell lysate and clinical specimens taken from the throat or nose was conducted on a rapid, easy-to-use and lightweight paper-based immunochromatographic strip (ICS) [209]. A sandwich immunoassay was performed using AuNP/gold enhancement substrate. The paper-based POC system was offered multiplexed detection from 5 μL sample with a LOD of 2.7×10^3^ plaque-forming units (PFU) and 2.7×10^4^ PFU for H1 and H3, which are surface glycoproteins on H1N1 and H3N2, respectively. In another paper-based detection system, DNA products of viruses such as MERS-CoV were detected [210]. Color change was observed with the help of nanoparticle aggregation resulting from the complex formation of target DNA and a specific probe called pyrrolidinyl peptide nucleic acid (acpcPNA). When there was no complementary DNA in the environment, an aggregation was formed by the interaction of silver nanoparticles (AgNPs) and acpcPNA, and thereby the color turned from yellow to red. This paper-based method was provided high-sensitive, rapid, and affordable colorimetric detection even in the presence of limited resource settings. LOD of this system was found as 1.53 nM for MERS-CoV. In another study, influenza A was detected by using a multiplex RT-LAMP with an immunochromatographic strip (ICS) containing sample field, conjugate pad, detection field, and absorbent pad (Fig. 2c) [211]. RT-LAMP products labeled with biotin were bound with AuNPs coated with streptavidin and IgG. Thereby, the gold nanoparticles were targeted to bind to the anti-IgG in the control line. Influenza A subtypes were amplified in 40 min and detected in 15 min down to 10 copies. It was also provided high accuracy of subtyping and detection from the nasal swab samples.

In a study of color-based LAMP virus detection, the nucleic acid products of the H1N1 virus were detected on a microfluidic chip controlled and monitored via a smartphone [212]. The extraction of the viral sample, cleansing, the application of the LAMP method, and the determination of the detection result were all included in the designed platform that was consisted of motors, Arduino control circuit, microfluidic chip, sensors for color and temperature monitoring, and modules for photo-interrupter and thermal control. The extraction of virus in microfluidic chip was procured via using functionalized magnetic beads. Colorimetric detection of LAMP product provided LOD of 3.2×10^−3^ HAU for H1N1 with an assay time of 40 min. Another colorimetric-based LAMP system was developed to detect multiple respiratory viruses in the microfluidic chip [23]. The extracted nucleic acids of H1N1, H3N2, H5N1, and H7N9 viruses were introduced in the microchannels and a color-based detection was provided via a real-time LAMP. It was shown that it could detect multiple respiratory viruses with a specificity of up to 100% and a sensitivity of up to 96% in clinical samples. LOD of 2–4 fg/μL can be obtained in this chip with a sample taken from the throat.

A DNA hydrogel formation–based rolling circle amplification system called DhITACT-TRail was reported for MERS-CoV detection [213]. By using a microfluidic system containing 3 different channels (sample, negative control, and positive control), it was possible to detect viral pathogens both with the naked eye and fluorescently within 2 h and 30 min, respectively. With the introduction of the RNA extract into the channel, the target base pair is attached to the primary base pairs on the surface and hybridization has occurred. In order to show the repeatability feature, analyzes were made with false serum and the detection limit was found as 6×10^7^ copies/device.

#### Others

Surface-enhanced Raman Scattering–based lateral flow immunoassay strip was developed for the detection of influenza A H1N1 and human adenoviruses using dual-layer Raman dye molecule conjugated Fe_3_O_4_@Ag magnetic tags [214]. LOD was found for the H1N1 50 PFU/mL and 20 PFU/mL for human adenovirus. This system is 2000 times more sensitive than the standard ICS. There are also THz plasmonic metasensor–based biosensors designed to detect SARS-COV-2 spike proteins in 80 min using toroidal resonances [215]. To increase the binding power of target biomolecules, functionalized AuNPs were added to multi-pixel metallic metasurfaces. These features allowed to reach a LOD value of about 4.2 fmol in 15 μL solution.

### Electrical detection techniques

Electrical detection has been used for direct detection of targets by using micro and nanofabricated electrodes, field-effect transistors, and semiconductor materials [216–219]. Detection can be achieved using electrical simple instrumentation with reduced response time and signal noise, and with increased sensitivity and portability [220].

The concentration of the influenza viruses collected from patients is very low. Therefore, it is required to amplify nucleic acid samples from viruses and PCR techniques are performed as a “gold standard” method [221]. RT-PCR chip was proposed for rapid detection of influenza A virus [222]. The chip was composed of four parts, which were the real-time reaction part, denaturation part, thermal cycles part, and pressurizing part. It offered a solution to the problem of generation of air bubbles encountered in continuous-flow microfluidic PCR systems by using a pressurizing channel located before the outlet. After reaction in the RT-PCR chip, the samples collected from the outlet were analyzed with disposable electrical printed (DEP) chips and electrochemical signals were measured using square wave voltammetry (SWV). Methylene Blue (MB) was used as an electrochemical hybridization indicator for electrochemical detection by carbon printed electrodes. Decrease in the amount of free electro-active MB was related to increased positive samples because MB bound to DNA. Thereby, MB reduction peak currents confirmed the detection of influenza A virus. Similarly, cDNA of the SARS-CoV-2 virus was detected using a low-cost silicon-based integrated Point-of-Need transducer (TriSilix) [223]. They used an electrical heater, temperature sensor, and electrochemical sensor on the chip for electrochemical-based detection with MB. A handheld potentiostat was used to obtain cyclic and square wave voltammograms and the system achieved detection of 1 pg of cDNA of SARS-CoV-2 with 40 min PCR cycling.

Influenza A virus detection system was also developed for distinguishing subtypes of the virus [221]. This system enabled amplification and detection processes with two modules. Basically, the first module (PCR module) was used to amplify nucleic acids from target strains (i.e., FluA and H1N1). Amplified target strains were then transported to the second module (sensing module). In this module, silicon nanowire (SiNW) immobilized with PNA probes for FluA or H1N1 was used for real-time electrical detection. In this system, label-free and multiplexed detection of subtypes of influenza A was achieved with the advantages of low sample consumption, high specificity, and sensitivity (20–30 fg/μL). SiNW field-effect transistor (FET)–based biosensor was developed for reusable and ultrasensitive virus detection with reversible surface functionalization strategy with a disulfide linker [224]. In the system, monoclonal antibody was used as a receptor for avian influenza virus detection with a LOD of 10^−17^ M.

Detection of DNA parts of avian influenza was achieved using a carbon nanotube (CNT)–based biosensor [225]. Two different chemiresistor-based sensors have been developed using semiconductor single-walled CNT and multi-walled CNT. CNTs immobilized with DNA probe allowed detection of the unlabeled virus sequence in less than 15 min. LOD of 2 pM and 20 pM were achieved for single-wall CNT and multi-walled CNT, respectively.

**Fig. 3:**
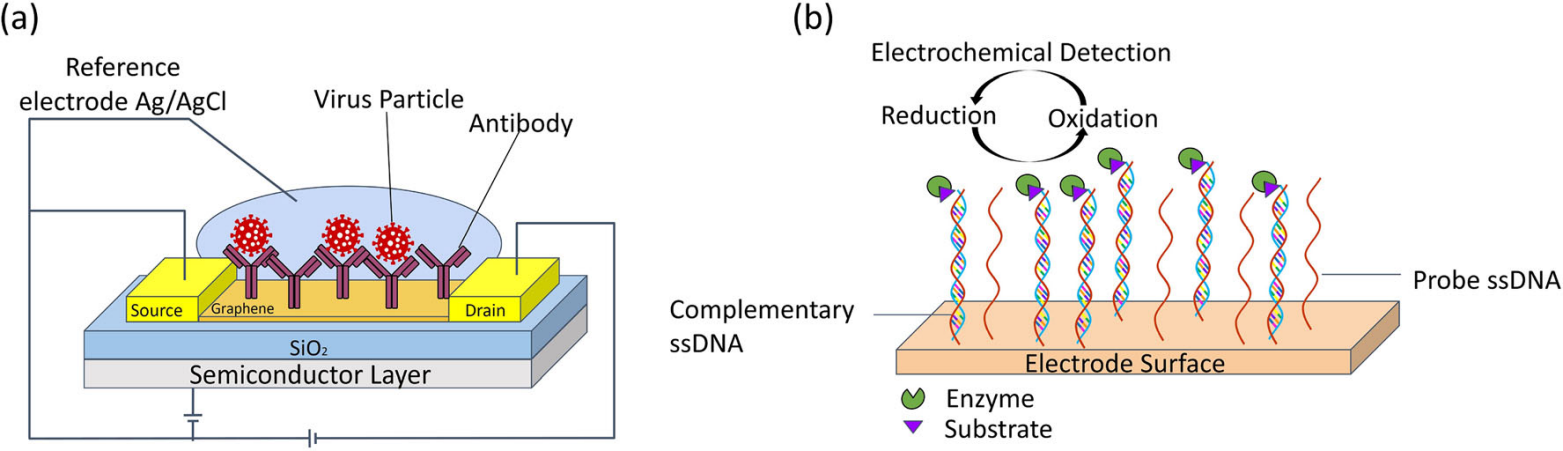
Illustrations of electrical-based methods for the detection of respiratory viruses or their products. **a** FET-based biosensor, **b** Electrochemical biosensors to detect DNA fragments

Reduced graphene oxide (rGO) transistor was used to detect gene parts of avian influenza [226]. On this device, three different immobilization techniques for DNA probes were evaluated and it was shown that the extended long capture probe immobilized by π-π stacking interaction has a higher sensitivity and stability compared to short capture probe (π-π interaction) and covalent immobilization via linker. The rGO transistor provided detection down to 5 pM with an assay time of 1 h. In another microfluidic chip using rGO sheets, H1N1 virus particles were detected without labels and sample preparation step [220]. The rGO surface was functionalized with monoclonal antibodies specific to the H1N1 virus. The electrochemical sensor detecting changes in chronoamperometric current showed a LOD of 0.5 PFU per milliliter. FET-based microfluidic device was also developed by coating graphene sheet with specific antibody for detection of SARS-CoV-2 spike protein (Fig. 3a) [227]. This device enabled rapid and selective detection of SARS-CoV-2 from directly nasopharyngeal swab suspended in universal transport medium with a LOD of 2.42 × 10^2^ copies/mL.

In another study, an alternative microfluidic biosensor platform was designed and developed to detect H1N1 [228]. Label-free detection of viruses was conducted through electron interaction and provided through covalent bond between DNA aptamers and functionalized conductive polymer microelectrodes. The developed platform measured the influenza A virus (H1N1) concentration in saliva and provided fast (less than 15 min), stable, sensitive, low-cost, and selective detection using impedance measurements. H1N1 virus particles were also detected using a combination of colorimetric immunoassay and electrochemical impedance spectroscopy (EIS) [229]. Filtration of the sample was achieved by using pads with large and small pores as 11 μm and 0.45 μm, respectively, and the antigen-antibody structures were concentrated on the conjugate pad. It has been stated that a more reliable detection is provided by using the combined method. H1N1 virus was detected down to 5 PFU/mL from PBS and saliva in 6 min via this sensor. Multiple detection of respiratory viruses (H1N1, H5N1, and H7N9) was also performed on an immunosensor [230]. The immunosensor chip enabling simultaneous and quantitative detection was constructed with the ZnO nanorods (NRs). Captured antibodies of three viruses were immobilized on ZnO NRs and sandwich ELISA procedure was conducted with a detection antibody labeled with HRP. Oxidation current resulting from the reaction of HRP and 3,3′,5,5′-tetramethylbenzidine (TMB) was used to detect influenza viruses down to 1 pg/mL (Fig. 3b) [230].

Detection of influenza A H9N2 was conducted with iron magnetic nanoparticles (MNPs) and AuNPs used for isolation and detection, respectively [231]. Antibody-conjugated MNPs formed a sandwich-like complex with Fetuin-AuNPs in the presence of target influenza virus. The virus detection was carried out based on gold catalysis and provided the rapid detection of H9N2 at a titer less than 16 HAU. An ultrasensitive device (eCovSens) using a screen-printed carbon electrode (SPCE) was developed for the detection of SARS-CoV-2 virus and compared with commercial potentiostat using fluorine-doped tin oxide (FTO) electrode, which was conducted by immobilization of AuNPs used for both immobilization of monoclonal antibody of SARS-CoV-2 and amplification of electrochemical signals [232]. This device offered a very rapid analysis down to 10–30 s using 20 μL sample volume of saliva with a LOD of 90 fM. Moreover, LOD is reduced to 10 fM in standard buffer. Even though the detection limits of the two systems were similar to commercial potentiostat, the developed device had advantages such as being portable and cost-effective. A microfluidic chip was also developed for influenza virus or influenza hemagglutinin detection [233]. Working method of the chip was based on two main steps that are isolation by paramagnetic beads and detection of the target electrochemically. Streptavidin–biotin affinity was utilized for isolated hemagglutinin on paramagnetic beads was labeled with cadmium sulfide (CdS) QDs and it was detected by voltammetry measuring changes in cadmium signals.

### Other detection techniques

Influenza A virus was detected via the novel surface acoustic wave (SAW) sensor capturing H1N1 viral antigen from liquid [234]. The SAW sensor surface was functionalized to immobilize HA antibodies for influenza A. The system includes a syringe pump system to control fluidic flow, microwave probes, and electrodes. The SAW sensor allowed sensitive detection down to 1 ng/mL antigen on the functionalized SAW surface using Love wave. Offering reliable detection, this approach could offer a fast and sensitive virus detection platform as a clinical diagnostic compared to commercial kits with low specificity.

Quartz-crystal microbalance (QCM)–based detection was achieved for the influenza virus RNA detection [235]. Target genome in the microfluidic channel was captured by PNA immobilized on QCM electrode. Gold colloid was used to increase virus RNA weight to increase sensitivity, and LOD was found as 10^6^ PFU/mL for this mass-based detection.

## Commercially available microfluidic solutions

Cepheid Xpert® Xpress (USA) is an automated molecular device for testing SARS-CoV-2, flu A, flu B, and RSV [236]. This device includes sample preparation, nucleic acid amplification, and detection in one. It has a simple design to use without professionals and gives positive results around 30 min and it has acquired Emergency Use Authorizations (EUA) from FDA. Roche cobas® (USA) is a real-time RT-PCR test designed for the qualitative detection of nucleic acids from SARS-CoV-2 [236]. The Roche platform is a batch-based platform that can perform multiple tests and contains 90 samples/runs every 90 min. Cobas® has been authorized by FDA under an EUA. Isothermal Microfluidic Chip Analyzer RTisochip™ (CHN) is developed for independent analysis of nucleic acid with high throughput, flexibility, high capacity, high accuracy, low cross-contamination, high efficiency, and multi-sample analysis [237]. It was developed by CapitalBio Technology Corporation and certificated by CE. This kit can detect SARS-CoV-2 and 5 other types of respiratory viruses. BioFire™ Filmarray® (USA) system is a microfluidic-based detection system that enables extraction, purification, and PCR amplification of nucleic acid in a single chip [22]. This device gives 19 viral and 4 bacterial target detection results around 1 h and it is so easy to use without pipetting or measuring the sample [238]. It was certificated by FDA, CE-IVD, and TGA and is currently used for fighting SARS-CoV-2. be.well™ by Alveo Technologies (USA) provides a rapid and easy-to-use detection platform to detect flu A/B, SARS-CoV-2, and RSV and to manage these viral diseases via be.well™ cloud-based applications. This platform is integrated with a smartphone and can provide a result within 30 min. The platform includes a microfluidic channel that can amplify nucleic acids in real-time [239]. Veredus Laboratories (SG) developed VereFlu Chip which is a nucleic acid–based test used for the qualitative detection, differentiation, and identification of influenza A (H1, H3, H5, H7, H9), influenza B viruses, and 2009 pandemic H1N1 in a single test [240]. Also, VereCoV™ Detection Kit was developed and used for the qualitative detection of the nucleic acid of MERS-CoV, SARS-CoV-1, and SARS-CoV-2 by integrating PCR and microarray. This detection kit can detect and differentiate 3 coronaviruses types in a single test for about 2 h [241]. Alere BinaxNOW® Influenza A & B Card developed by Abbott Company (USA) is an immunochromatographic assay for the rapid detection of influenza A and B [242]. It enables simultaneous, qualitative detection for two types of influenza viruses in just 15 min by using samples from nasal/nasopharyngeal swab and nasal wash/aspirate. Current technology developed by Abbott Company for detection of influenza A and B is the ID NOW™ which is an isothermal nucleic acid amplification-based POC device [243]. It gives a negative result within 13 min and a positive result within 5 min from swab samples. The company has also developed ID NOW™ RSV and ID NOW™ COVID-19 devices for nucleic acid detection of RSV and SARS-CoV-2, respectively [244, 245]. Quidel Corp. developed QuickVue® (USA) test which is a rapid lateral flow immunoassay, and it can detect influenza, adenovirus, bordetella, SARS-CoV-2, RSV, and some other respiratory viruses [246–248]. The QuickVue® influenza test kit gives results in 10 min [249], and it takes 15 min in the RSV test [250]. The kit has relatively low sensitivity compared to its high specificity [247, 251]. Also, the results of the statistical measures of the performance change by specimen collection method and age group [249, 251]. Besides these, there are so many different tests and devices for the detection of SARS-CoV-2 and the other respiratory viruses [252].

## Challenges of virus detection

Key point of the successful microfluidic chips and sensors design is related with the main eight performance factors which are stability, sensitivity, selectivity, detection range, response time, repeatability, LOD, and lifetime [253, 254]. Low detection limit is required to detect infection in early stages for rapid and effective treatment. Multiple detection systems are important for specific, sensitive, and accurate diagnosis of the target virus or its subtypes [22]. Also, more than one virus infection at the same time can be seen in some cases. In this point, multiple detection is more advantageous to understand severity of the illnesses in people with different viral loading and compare the impact of the viruses in mixed infection [255]. Hence, selectivity is a crucial parameter in these systems. Microfluidic platforms should also offer portable, low-cost, easy-to-use, and fast detection schemes for rapid and point-of-care assessment of respiratory viruses. Storage conditions and operational procedures of these platforms need to be easy-to-handle for increasing access to rapid diagnosis even in rural areas. In microfluidic devices, environment and external influences have an effect on detection of virus components or virus itself. Temperature has a primary effect on many microfluidic devices. It severely affects detection methods using PCR and LAMP [222]. Temperature should be controlled for sensitive and efficient detection. Heating is also a factor that must be controlled in designed devices, as it affects the electrochemical interaction, optical signals, microwaves, and the speed of molecular reactions in the sample [256]. Mechanical effects are also factors affecting the detection in microfluidic devices. Pressure [257], fluid dynamics [258], mixing effect [259], etc. may change the efficiency of the detection. External electric [260] and magnetic [204] fields can also affect the measurement techniques and detection efficiency of microfluidic devices using magnetic, electronic, and electrochemical techniques. Therefore, microfluidics devices should be tested for external effects also so that precautions could be taken to eliminate these adverse effects on the detection performance.

There are different protocols and methods that should be used to keep the signal levels at the highest level in the determination of respiratory tract viruses. The most important steps are specimen collection, extraction, amplification, and measuring signal levels for sensitive detection [11]. Although labeled detection methods offer sensitive direct detection, labeling process increases assay time and cost [261].

The process of collecting samples/specimens from different locations of the body of respiratory viruses from human varies from virus to virus [34, 262]. Respiratory virus samples can be collected from feces, serum, sputum, bronchial lavage, nasal swab, nasal brush, nasal wash, blood, tear, oropharyngeal swab, pharyngeal swab, nasopharyngeal wash, nasopharyngeal aspirate, conjunctival swab, lung biopsy, etc. [263–268]. However, collection, transport, and storage conditions of the specimen could affect detection sensitivity [264]. Sample preparation, collection, transport, and detection can be performed on a chip level using microfluidic technology [4, 269].

Samples taken for the nucleic acid–based detection of respiratory tract viruses are subjected to an extraction process so that unwanted substances are removed, and the unity of viral nucleic acids is preserved. Nowadays, different methods are used for the extraction of nucleic acids from different sources [270]. These methods include phenol-chloroform extraction, solid-phase extraction methods, the bead-based system providing extraction with magnetic control, and the filtration method provided with a filter paper. The phenol-chloroform extraction method, which can damage nucleic acids and create harmful waste after extraction, is low-cost but requires experienced technicians [271, 272]. The solid-phase extraction method, based on column and membrane, is a rapid and simpler method, but requires multiple centrifugations and laboratory devices for extraction steps [11, 273, 274]. In the magnetic bead-based extraction method, which is based on the use of functionalized magnetic beads, nucleic acids bind magnetic beads and allows isolation of nucleic acids [270, 275]. This method can require multiple washing processes [11, 276]. It can be also conducted in microfluidic platforms using functionalized magnetic beads [277]. In extraction by filtration method, commercially available paper and membrane structures allow isolation of nucleic acids [278, 279]. This method, which is quite affordable and simple, is also suitable for use in microfluidic platforms.

Nucleic acid amplification methods for the virus detection have many advantages over serological tests. Nucleic acid amplification offers sensitive detection because produced antibodies are not able to be detected at the early phase of the virus [11]. PCR, LAMP, recombinase polymerase amplification (RPA), helicase-dependent amplification (HDA), exponential amplification reaction (EXPAR), nucleic acid sequence–based amplification (NASBA), transcription-mediated amplification (TMA), and ligase chain reaction (LCR) are the types of amplification methods [280–282]. PCR is performed with a number of temperature cycles including denaturation, annealing, and extension by the help of DNA polymerase [269]. LAMP is performed at fixed temperature (65 °C); 10^9^ amplicon production is achieved in less than 1 h. LAMP can be easily integrated to microfluidic systems and the detection of amplified products can be conducted even with naked eye inspection [269, 283]. Due to low isothermal incubation temperature, sensitivity, and high tolerance to impurities in sample, RPA has significant utilization in microfluidics [284]. HDA uses ds-DNA unwinding activity of helicase to separate strand and produced amplicons by strand displacing DNA polymerase [285]. HDA is also one of the mostly used amplification techniques in microfluidics due to its simplicity and sensitivity [269]. TMA and NASBA amplify RNA using RNase and RNA polymerase activity. RNase digests the RNA and RNA polymerase produces copies. NASBA is performed isothermally thus, does not require a thermocycler. In LCR, ds-DNA is denatured, and primers anneal to each strand, then ligation occurs by DNA ligase [286]. For nucleic acid amplifications, microfluidic platforms offer many advantages, such as simple heat transfers due to their small size, rapid detection, portability, high specificity, and automation [287, 288].

Immobilization of capturing agents is also the key point for the desired detection [289]. The capturing agents can be grafted on surfaces using physical adsorption, covalent attachment, bioaffinity immobilization, streptavidin-biotin immobilization, etc. depending on the chip surface and properties of capturing agents [290–292]. Silicon is mostly used material for the integrated circuit devices and microelectromechanical systems. However, opaqueness of silicon can limit the optical imaging and requirement of cleanroom environment can increase the cost of fabrication [293]. Glass substrates are well-suited generally for optical-based detection methods but it has some disadvantages such as brittle and not easily processed [294]. PDMS is one of the most favored materials due to its low-cost, flexible, and transparent structure [295]. PDMS can be easily fabricated using a soft lithography technique where microfabricated or even 3D printed molds can be used [20]. The limited resistance to organic solvents and challenges of hydrophobic structure of PDMS limits its utilization [294]. Moreover, hybrid fabrication technique utilizing bonding of 3D printed structures with surface functionalizes surfaces enhances the usability of 3D printed technology in microfluidic-based sensing methods [20]. For direct detection of intact target viruses or virus residues, antibody, aptamer, DNA probe, and PNA probe are utilized in microfluidic devices as capturing agents of targets [294, 296–298]. Recently, aptamers are used instead of antibodies due to their less sensitivity to environmental factors [292].

## Summary and Outlook

During viral infection, expression of IgM antibodies takes place after 3–7 days of infection, and IgG antibodies can be detectable after 8 days which is the main challenge for the viral detection [299]. The most disadvantage of IgG/IgM detection is that produced antibodies can react with cross-reactive antibodies that cause false positives of patients without SARS-CoV-2 [299]. According to WHO recommendation, serological testing should be used to understand if someone has ever been infected and assessment of immunity, not for clinical diagnostics [300]. On the other hand, even if the immune system is not yet activated, viruses and their contents can be detected directly for early diagnosis. Thus, direct detection methods are more efficient for the precise virus detection.

Saliva, swab samples, nasal fluid, and blood are analyzed for the direct detection of respiratory viruses by various detection methods. Conventional methods for the detection of viruses such as PCR and ELISA have some disadvantages such as the requirement for trained personnel as well as costly and complex equipment. Microfluidic technologies offer accurate and specific methods for direct detection of respiratory tract viruses. Direct detection techniques play an important role for the diagnosis of respiratory diseases with high specificity and sensitivity. Microfluidic systems enable to reduce sample consumption, assay time, and cost of tests without compromising form assay sensitivity. Hence, microfluidic-based detection technologies are beneficial in pandemic and epidemic situations by providing rapid, portable, and accurate detection of viruses.

The use of microfluidic technologies provides many advantages as compared with conventional methods. Since microfluidic devices are time- and cost-effective devices, preference of these devices is increased over time. Also, detection efficiencies increase through miniaturization and the use of small volumes [301, 302]. Microfluidic systems for detection of respiratory virus detection are summarized in Table 1. Each detection method has different advantages. For instance, absorbance-based detection can reach low LOD. Fluorescent-based detection focuses on the direct detection of virus particles by reducing analysis time. Although colorimetric-based methods have a longer analysis time compared to other optical methods, they allow naked-eye detection. Many methods have been applied in electrical detection and their general features are short analysis time with a low LOD. Thus, different detection methods can be preferred when considering extraction, target analyte, LOD, and analysis time. Since electrical detection can provide portable and label-free detection, this detection strategy can be a good candidate for rapid virus detection on-site. Since amplification and labeling strategies can increase assay time and cost, new nanomaterials, or nanostructures could be utilize as sensor elements to enhance sensitivity of electrical detection methods further.

**Table 1:** Summary of microfluidic systems used for detection of respiratory virus detection

Reference	Detection method	Extraction	Virus	Target analyte	LOD	Assay time
188	Absorbance	–	SARS-CoV-2	N-protein	10^–18^ M	15 min
187	Absorbance	–	SARS-CoV-2	Surface proteins	100 units/mL	~1 h
189	Absorbance	–	H1N1	Virus particle	2.9 × 10^3^ PFU/mL	1 h
201	LSPR	–	SARS-CoV-2	AuNIs with complementary DNA receptors	0.22 pM	800 s
74	SPR	Nucleic acid extraction kit	Influ A, Influ B, PIV 1, PIV 2, PIV 3, RSV, SARS, ADV, H1N1	Oligonucleotides	5 nM for Influ A, 1 nM for Influ B, 1 nM for PIV 1, 2.5 nM for PIV 2, 3.5 nM for PIV 3, 3 nM for RSV, 0.5 nM for ADV, 2 nM for SARA, 3 nM for H1N1	~30 min
202	SPR	–	H1N1	Nucleoprotein	NR	NR
203	Fluorescent	On-chip	SARS-CoV-2	Nucleic acid	10 copies/μL	30 min
204	Fluorescent	–	H1N1, H3N2 and H9N2	cDNA	0.21 nM for H1N1, 0.16 nM for H3N2, 0.12 nM for H9N2	80 min
205	Fluorescent	Yes	H1N1, H3N2 and Influenza B	Virus particle	3.2 HAU	20 min
206	Fluorescent	–	H1N1, H3N2	Virus particle	5 × 10^−4^ HAU	15 min
207	Fluorescent	–	H9N2, H1N1, H7N9	Virus particle	0.02 pg/mL	NR
209	Colorimetric	–	H1N1 and H3N2	Proteins inside and outside of the influenza virus	2.7 × 10^3^ PFU/assay for H1 and 2.7 × 10^4^ PFU/assay for H3	1 h
210	Colorimetric	Experimental approach	MERS-CoV	DNA parts	1.53 nM	NR
211	Colorimetric	Nucleic acid extraction kit	H1N1, H3N2, and H5N1	RT-LAMP products	10 copies of viral RNA	55 min
212	Colorimetric	Bead-based extraction	H1N1	RNA or DNA	3.2 × 10^−3^ HAU/reaction	40 min
23	Colorimetric	Bead-based extraction	H1N1, H3N2, H5N1, and H7N9	Nucleic acids	2–4 fg/μL	30 min
213	Colorimetric		MERS-CoV	RNA	6×10^7^ copies/device	2 h
214	Surface enhanced raman scattering	–	H1N1 and human adenovirus	Virus particle	50 PFU/mL for H1N1 and 20 PFU/mL for human adenovirus	NR
215	Plasmonic	–	SARS-CoV-2	Spike protein	~0.28 nM	~80 min
222	Electrical	Nucleic acid extraction kit	Influenza A	PCR amplicon	5.36 × 10^3^ copies/μL	~15 min
223	Electrical	DNA extraction kit	SARS-CoV-2	cDNA	1 pg	~40 min
221	Electrical	Gel extraction kit	H1N1 and H3N2	DNA	20–30 fg/μL	NR
224	Electrical	–	H5N2	Virus particle	10–17 M	NR
225	Electrical	Yes	H5N1	DNA parts	2 pM	15 min
226	Electrical	–	H5N1	DNA parts	5 pM	1 h
220	Electrical	–	H1N1	Virus particle	0.5 PFU/mL	NR
227	Electrical	–	SARS-CoV-2	Spike protein	2.42 × 10^2^ copies/mL	> 1 min
229	Electrical	–	H1N1	Virus particle	5 PFU/mL	6 min
230	Electrical	–	H1N1, H5N1 and H7N9	Virus particle	1 pg/mL of each virus	NR
231	Electrical	–	H9N2	Virus particle	16 HAU	160 s
232	Electrical	–	SARS-CoV-2	Spike protein	90 fM in spiked saliva sample, 10 fM in standard buffer	10–30 s
233	Electrical	–	H5N1	Surface antigen (hemagglutinin)	–	45 min
234	Acoustic	–	H1N1	HA antigen	1 ng/mL	NR
235	Mass	–	Influenza	RNA	10^6^ PFU/mL	NR

*NR* not reported

All specified commercial diagnostic kits and medical devices must obtain conformity certificates from the approving authorities (FDA, NMPA, etc.). Microfluidic diagnostic devices can be commercialized in accordance with specified quality parameters and performance criteria determined by these approving institutions. For this reason, the features that distinguish commercial products from each other are generally the diagnostic method, speed of diagnosis, LOD, diagnostic capacity, etc. When we look at commercial products in the detection of respiratory tract viruses, it is shown that products where all assay stages such as sample preparation, extraction, and amplification are performed on chip are more efficient. Moreover, it is of great importance that commercial devices can make rapid and also multiplex detections.

Microfluidic technologies can offer accurate and sensitive detection of respiratory tract viruses. Direct detection techniques enhance the specificity and sensitivity of virus detection. This review focused on the direct detection technologies developed in microfluidic systems for the detection of respiratory. These techniques could reduce sample volume, assay time, and test cost and they could also provide portable, rapid, and sensitive detection of respiratory viruses that could be used for controlling outbreaks with rapid assessment of viruses.
